# Direct No-Shave Follicular Unit Excision (DNS FUE): A Modified Technique and Case Series

**DOI:** 10.7759/cureus.102271

**Published:** 2026-01-25

**Authors:** Renan Brigante, Alan Wells

**Affiliations:** 1 Department of Surgery, Grajau General Hospital, São Paulo, BRA; 2 Department of Plastic and Reconstructive Surgery, Hospital das Clínicas of the Faculty of Medicine of the University of São Paulo, São Paulo, BRA

**Keywords:** direct no-shave follicular unit extraction, dns fue hair transplant, follicular unit excision, follicular unit extraction, fue hair transplant, hair transplant, long hair transplant

## Abstract

Hair transplantation has advanced substantially over recent decades, with follicular unit excision (FUE) becoming a widely adopted minimally invasive alternative to traditional strip harvesting. Despite these developments, the need to shave the donor region remains a significant aesthetic and psychosocial barrier for patients who prioritize procedural discretion and a rapid return to daily activities. In this technical report, we describe a refined direct no-shave follicular unit excision (DNS FUE) method designed to eliminate donor-area shaving while preserving graft integrity and donor-site density. We applied this technique to 10 patients with Norwood Class IV and Ludwig Class 2 androgenetic alopecia, yielding an average of 4,600 extracted follicular units per procedure. The approach utilizes the Trivellini Flared Ring punch under intravenous sedation. A central refinement in our method is the incorporation of this trumpet-shaped punch, which was introduced with the intention to improve axial stability, reduce oscillation during hair-shaft engagement, and consequently minimize follicular trauma. Following the adoption of this modification, we observed a mean transection rate of 3%. The combination of low transection rates and high patient-reported satisfaction suggests that this modified approach to DNS FUE might be an aesthetically favorable option for appropriately selected candidates requiring discreet, high-density follicular unit excision. Future studies with larger sample sizes and a case-control design are essential to validate these observations.

## Introduction

Hair transplantation has undergone major refinement over the past three decades, evolving from follicular unit transplantation (FUT), where a strip of scalp is removed and dissected, to highly natural outcomes through follicular unit-based techniques [1]. Among these, follicular unit excision (FUE) represents a significant advancement by avoiding linear scarring and reducing donor-site morbidity compared to the FUT method [1]. However, conventional FUE typically requires shaving the donor area, creating aesthetic, social, and psychological barriers for patients who prefer to keep their procedure discreet [2]. Indeed, the requirement to shave the donor region remains one of the primary reasons patients hesitate to choose FUE [3].

To overcome this limitation, long-hair (LH) and no-shave FUE variations were developed, allowing graft harvesting without trimming the donor zone. Despite their conceptual advantages, early implementations presented important technical and procedural challenges. Schambach (2020) directly compared LH-FUE with shaved FUE and demonstrated that, while the long-hair approach offered superior concealment, it required advanced surgical expertise and nearly doubled operative time [4]. The technique also resulted in an 8% increase in partial transection, attributed to reduced visualization and interference from long hair shafts during excision, factors that made early LH-FUE technically demanding, time-consuming, and highly operator-dependent [4].

More recently, Umar et al. (2023) introduced a no-shave FUE approach using a skin-responsive device engineered to adapt dynamically to variations in scalp firmness and thickness [5]. This innovation reduced total transection rates to 2.2-4.3% and achieved a mean extraction speed of 440 grafts per hour [5]. Furthermore, according to the authors, the adaptive punch mechanics increased procedural consistency and surgeon willingness to perform no-shave FUE, with self-reported readiness rising from 1.25 to 4.20 on a five-point scale [5]. Despite these advances, such systems rely on specialized equipment that is not widely accessible, and challenges remain regarding mechanical instability when long hair shafts are severed before full follicular isolation.

Building upon these developments, we propose a refined direct no-shave FUE (DNS FUE) technique that integrates the discretion of LH-FUE with the mechanical precision of responsive systems, while maintaining practical accessibility for surgeons in Brazil and other countries. A key refinement is the use of the Trivellini Flared punch, which has a trumpet-shaped tip that keeps the follicle units away from the cutting edge, thus functioning as a centering guide. This geometry seems to stabilize the punch axis as it traverses the hair shaft and dermis, minimizing oscillation, improving axial alignment, and was hypothesized to reduce both partial and total transection. We report our clinical experience following the implementation of this modification in a consecutive series of 10 patients.

## Technical report

We hereby describe the methodology, patient selection, and clinical outcomes of the DNS FUE technique based on a series of 10 cases. Eligible patients included five female patients with moderate to advanced androgenetic alopecia (Ludwig scale II and III) and five male patients with advanced androgenetic alopecia (Norwood stages IV and V). All patients continued their oral medical therapies without interruption, and each had been on treatment for at least three months prior to surgery. Patient demographics and preoperative therapies are summarized in Table 1.

**Table 1:** Patient demographics, treatment history, operative data, and clinical outcomes F = female; M = male; PO = oral; OD = once daily; mg = milligrams; TTR = total transection rate. Seven-point scale: +2 = moderately improved; +3 = greatly improved.​​​​​

Patient	Gender	Age (y)	Alopecia class	Preoperative treatments (OD)	Total grafts (x1000)	TTR (%)	Op time (h)	Grafts/h	Seven-point photo scale	Satisfaction score (1-10)
1	F	34	Ludwig II	Spironolactone 100 mg; minoxidil 1.0 mg	4.2	2.8	7.5	560	+3	9
2	F	45	Ludwig III	Spironolactone 50 mg; minoxidil 1.5 mg	4.85	3.2	9.0	539	+3	10
3	F	52	Ludwig II	Minoxidil 1.25 mg	4.4	2.5	8.0	550	+2	9
4	F	29	Ludwig II	Spironolactone 100 mg; minoxidil 0.5 mg	4.35	3.1	7.0	621	+2	8
5	F	61	Ludwig III	Dutasteride 0.5 mg; minoxidil 1.5 mg	4.7	3.4	9.5	495	+3	10
6	M	56	Norwood IV	Dutasteride 0.5 mg; minoxidil 2.5 mg	4.6	2.9	8.5	541	+3	10
7	M	49	Norwood V	Dutasteride 0.5 mg; minoxidil 2.5 mg	5.1	3.5	10.0	510	+3	9
8	M	62	Norwood IV	Dutasteride 0.5 mg; minoxidil 2.5 mg	4.5	3.0	8.0	563	+3	10
9	M	38	Norwood V	Dutasteride 0.5 mg; minoxidil 2.5 mg	4.9	3.1	9.5	516	+2	9
10	M	50	Norwood IV	Dutasteride 0.5 mg; minoxidil 1.5 mg	4.4	2.5	7.5	587	+3	10
Mean	-	47.6	-	-	4.6	3.0	8.45	548	+2.7	9.4

**Figure 1:**
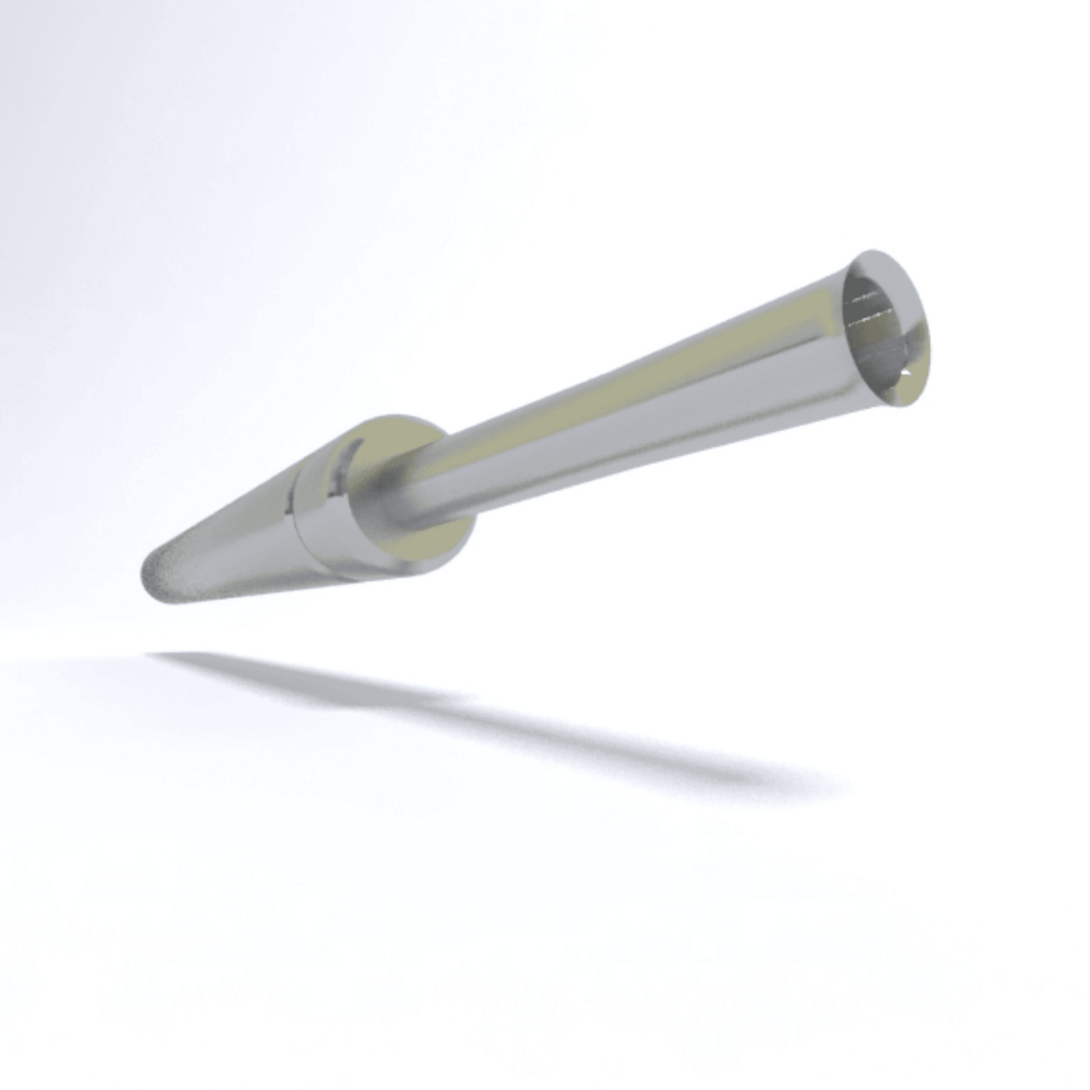
Trivellini Flared punch. Source: [6]

The procedure employed the Trivellini trumpet-shaped punch (Figure 1) for follicular unit excision. All procedures were performed under intravenous sedation administered and monitored by a licensed anesthesiologist. A multidisciplinary team of six to seven trained professionals assisted with excision, graft handling, and implantation. To minimize follicular ischemia, the excision phase was limited to a maximum of four hours and 30 minutes. Implantation was carried out using 0.64 and 0.8 mm implanters, allowing precise control of graft angulation, depth, and direction. The total procedure time ranged from seven to 10 hours.

Across the cohort, the mean number of follicular units extracted per procedure was 4,600. Following the introduction of the trumpet-shaped punch, the average total transection rate was 3%. Transection was defined as any structural disruption of a follicular unit in which the hair shaft or dermal papilla was partially or completely severed during punch excision. Total transection referred to complete separation of the follicular bulb or dermal papilla from the shaft, rendering the graft nonviable, whereas partial transection denoted incomplete injury where one or more shafts within the unit were cut but the follicular base remained intact. Transection rates were assessed in real time during the excision phase: each graft was examined immediately after excision under ×5-×10 loupe magnification by a trained technician and categorized as intact, partially transected, or totally transected. The total transection rate (TTR) was calculated as the number of totally transected grafts divided by the total number of extracted grafts, and the overall transection rate (OTR) included both partially and totally transected grafts. All evaluations were performed by the same surgical team to minimize inter-observer variability. Another key operative advantage of the no-shave technique was the ability to continuously visualize donor density during excision, enabling more strategic spacing of excisions, optimizing donor preservation, and reducing the risk of overharvesting.

**Figure 2:**
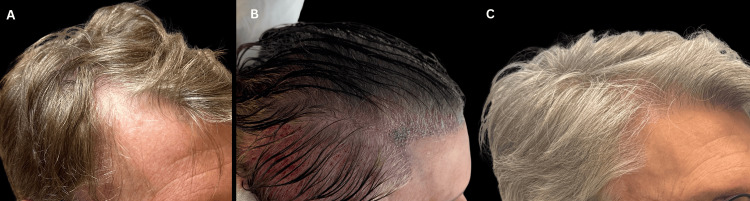
Clinical progression of a single male patient from the study cohort undergoing direct no-shave follicular unit excision (DNS FUE). (A) Preoperative view showing androgenetic alopecia in the right frontotemporal region. (B) Intraoperative appearance of the same recipient area immediately following graft implantation. (C) Six-month postoperative follow-up demonstrating hair growth and improved density.

At six to eight months postoperatively, all patients demonstrated significant clinical improvement as documented by the standardized seven-point rating score using scalp photographs and reported in Table 1, accompanied by uniformly high satisfaction rates. On a standardized 1-10 numerical rating scale, all 10 participants reported satisfaction scores of 8 to 10, indicating consistently favorable subjective outcomes (Figure 2). Overall, the DNS FUE technique appears to be feasible, and early results suggest that it is reproducible and effective for appropriately selected patients.

## Discussion

This case series presents outcomes from 10 patients who underwent a modified DNS FUE approach, demonstrating a low mean total transection rate of 3% following the introduction of a trumpet-shaped, long-hair punch. Although direct comparison across studies requires consistent definitions of transection, this value aligns closely with the 2.2-4.3% graft transection rate (GTR) reported by Umar et al. (2023) using a skin-responsive no-shave device [5]. By contrast, Schambach et al. (2020) demonstrated that while total transection rates in LH-FUE were comparable to those of conventional shaved FUE, partial transection rates were approximately 8% higher, highlighting the technical difficulty posed by interference from long hair shafts during dissection [4].

These technical challenges reflect the historical evolution of FUE. Traditional FUE required shaving the full donor area or large segments of it, creating significant aesthetic barriers for patients seeking procedural discretion [7]. Early attempts to minimize shaving by harvesting through small shaved patches proved suboptimal. When surrounding hair was long, a single patch might be concealed; however, shorter hair required multiple small islands, each prone to visibility issues [7]. Concentrated excision within these confined regions frequently produced dense clusters of hypopigmented extraction points, which many patients found more conspicuous than the strip-scarring patterns they sought to avoid [7]. These limitations contributed to the shift toward fully non-shaven FUE methods [7].

Contemporary LH-FUE techniques often employ grooved or slotted punches to reduce hair-shaft breakage and improve engagement with long hair; however, these systems require advanced technical skill and typically slow the procedure [4]. The only published large-scale evaluation of a skin-responsive LH-FUE method without recess-tipped punches is the multicenter study by Umar et al. (2023), which reported GTR values of 2.2-4.3% and a shaft break rate of 12.2%, with an average graft-excision speed of 440 grafts/hour using 18G and 19G punches [5]. Higher transection and break rates correlated with thicker, firmer scalps and higher SFS difficulty classes, rather than hair curliness [5]. Patient satisfaction was high, and the surgeon's willingness to perform no-shave LH-FUE increased substantially after adopting the device [5].

The guiding “trumpet punch” used in our DNS FUE surgical approach aligns with the same principles of mechanically stabilizing the punch during long-hair engagement. By improving axial alignment and reducing oscillation at the moment of hair-shaft entry, the punch helps mitigate a major source of partial and total transection previously observed in long-hair and patch-based techniques [4,5]. Collectively, these observations underscore that punch geometry and extraction mechanics are central determinants of success in no-shave FUE. These refinements directly address the aesthetic and technical limitations noted in earlier iterations of the procedure and support ongoing innovation in minimally invasive hair restoration.

## Conclusions

The DNS FUE technique, enhanced by the use of a trumpet-shaped punch, in our case, the Trivellini Flared punch, appears to be a technically feasible and effective option for hair restoration in appropriately selected patients. The punch’s stabilizing geometry is designed to reduce oscillation and improve axial alignment and was associated with a low total transection rate across our cases. Although these findings are encouraging, our study’s small sample size and lack of a control group limit the strength of the conclusions. Larger, controlled comparative studies are warranted to more definitively assess the technique’s efficacy, reproducibility, and long-term donor preservation relative to other no-shave and LH-FUE methods.
